# Separating variability in healthcare practice patterns from random error

**DOI:** 10.1177/0962280217754230

**Published:** 2018-01-31

**Authors:** Laine E Thomas, Phillip J Schulte

**Affiliations:** 1 Department of Biostatistics and Bioinformatics, Duke University, Durham, NC, USA; 2 Department of Health Sciences Research, Mayo Clinic, Rochester, MN, USA

**Keywords:** Provider variation, practice patterns, hierarchical data, measurement error, moment-adjusted imputation

## Abstract

Improving the quality of care that patients receive is a major focus of clinical research, particularly in the setting of cardiovascular hospitalization. Quality improvement studies seek to estimate and visualize the degree of variability in dichotomous treatment patterns and outcomes across different providers, whereby naive techniques either over-estimate or under-estimate the actual degree of variation. Various statistical methods have been proposed for similar applications including (1) the Gaussian hierarchical model, (2) the semi-parametric Bayesian hierarchical model with a Dirichlet process prior and (3) the non-parametric empirical Bayes approach of smoothing by roughening. Alternatively, we propose that a recently developed method for density estimation in the presence of measurement error, moment-adjusted imputation, can be adapted for this problem. The methods are compared by an extensive simulation study. In the present context, we find that the Bayesian methods are sensitive to the choice of prior and tuning parameters, whereas moment-adjusted imputation performs well with modest sample size requirements. The alternative approaches are applied to identify disparities in the receipt of early physician follow-up after myocardial infarction across 225 hospitals in the CRUSADE registry.

## 1 Introduction

Clinical studies frequently seek to improve the quality of care provided to patients by identifying discrepancies between providers. Provider units may be individual physicians, clinics, or hospitals. The first step is to establish that systematic variation exists and is of sufficient magnitude to justify a detailed investigation. Wide variation in outcomes supports the hypothesis that institutional factors play a role in affecting outcomes. Variation in treatment patterns suggests that the quality of care may be improved by identifying the practices of best performing institutions and adopting them widely. If the discrepancies between providers are small, improvement in quality will most likely come from new developments in care, rather than existing models. Consequently, recent publications emphasize conclusions about the magnitude in variation across providers: “the degree of variability in clinical practice we observed represents a potential quality improvement opportunity”;^1^ “in-hospital major bleeding rates varied widely across hospitals”;^2^ and “ICU admission rates for heart failure (HF) varied markedly across hospitals”.^3^

**Figure 1.**
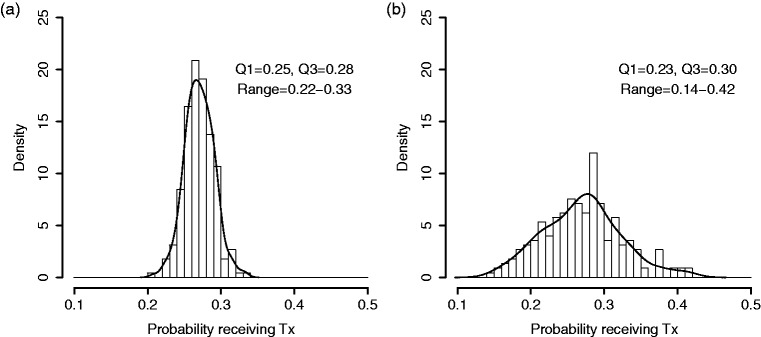
Comparison of distributions across providers. (a) Simulated distribution of true proportions from 225 hypothetical hospitals in which the true probability of receiving treatment ranges between 20 to 33%. (b) Simulated distribution of hospital-specific sample proportions, from the same hospitals, where the number of patients-per-hospital is randomly selected from the observed hospital sizes in the CRUSADE registry.

These data arise hierarchically, with a sample of providers, and within them, a sample of patients who experience a binary treatment or outcome. The goal is to quantify variation at the provider level in the proportion of patients who experience the outcome. Such data are commonly modeled by hierarchical logistic regression with a Gaussian random effect for provider whereby the provider-level variance can be estimated. This variance, measured on the log-odds scale, has limited the clinical interpretability, and the investigators prefer to visualize the variation in provider probabilities on an absolute scale. Therefore, numerous examples in the cardiovascular literature include a histogram of provider-specific sample proportions.^3–5^ For example, Hess et al.^5^ sought to identify disparities in the receipt of early follow-up (within seven days) with a physician after myocardial infarction. Across 225 hospitals enrolled in the CRUSADE registry, the hospital-specific sample proportions of early follow-up ranged from 2.6% to 51.6% (interquartile (IQR) range: 17.7%–29.1%). Such observed variation in sample proportions is predictably larger than underlying variation in provider probabilities, as each sample proportion is subject to sampling error. To address this, the authors followed a common convention of excluding sites with less than 25 patients.^3–5^ The convention is not sufficient. As an illustration, Figure 1(a) shows a hypothetical distribution of proportions from 225 hospitals in which the probability of receiving treatment is randomly generated and ranges between 20% to 33%, with an interquartile range of 3%. Figure 1(b) shows raw proportions that are observed if patient outcomes are sampled from these hospitals, with the number of patients-per-hospital randomly selected from the observed hospital sizes in the CRUSADE registry. As can be seen by comparing Figure 1(a) and (b), the raw proportions overstate the variation by nearly three-fold.

There is a large body of literature on methods for hospital monitoring and profiling,^6,7^ including recent recommendations from a panel of experts commissioned by the Committee of Presidents of Statistical Societies.^8^ These sources review, in detail, the alternative methods for profiling individual hospitals. Hierarchical models and shrinkage estimators are advocated as a strategy to remove the excessive sampling error in estimates for small sites and are widely used in the medical literature.^6–8^ However, the objective of making predictions for individual sites differs from the specific goal of estimating the magnitude of variation across sites. For the latter objective, estimating potentially hundreds of nuisance parameters (the performance of each hospital) and then pulling them back together to form a distribution results in more error than is necessary, in the form of either over-dispersion (exhibited in the raw proportions above) or shrinkage toward the mean. Although provider-specific sample proportions are over-dispersed, the distribution of shrinkage estimators is too narrow relative to the distribution of true provider performance.^8–10^

The fact that naive methods remain common in provider-variation studies may reflect that (1) the magnitude of the problem is not well appreciated and (2) the relative advantages and/or limitations of alternative methods are not clear in this context. Although several approaches for estimating and illustrating cluster-level variability in hierarchical data have been proposed, prior studies have focused on different targets of inference,^11,12^ continuous outcomes,^11,13–16^ or large cluster sizes.^12,13^

We consider three existing methods for binary outcomes: (1) Gaussian hierarchical models (GHM), (2) Bayesian semi-parametric beta-binomial model with a Dirichlet process (DP) prior,^17^ and (3) non-parametric empirical Bayes (EB), smoothing by roughening (SBR).^13^ Alternatively, we propose that a recently developed method for density estimation in the presence of measurement error, moment-adjusted imputation (MAI), can be adapted for this problem.^18^ In Section 2, we describe the methods under consideration. In Section 3, the methods are evaluated by simulation across a range of conditions that are motivated by clinical examples. Finally, the methods are applied and interpreted in the CRUSADE example of early physician follow-up.

## 2 Methods

For clarity of exposition, suppose we are interested in studying treatment variation across hospitals. Other binomial outcomes of clusters could easily be substituted. Let i=1,2,…,N represent the *i*th hospital and $j=1,2,\ldots ,n_{i}$ represent the *j*th patient from the *i*th hospital, where *N* is the number of hospitals and *n_i_* is the number of patients at the *i*th hospital. Denote *Y_ij_* as a binary indicator for whether the *j*th patient from the *i*th hospital receives treatment, taking a value of 1 if that patient received treatment, and 0 otherwise. The sum of *Y_ij_* over patients within the same hospital is denoted *Y_i_*. *Y_i_* may be viewed as independent, binomial counts with a hospital-specific probability of receiving treatment, *p_i_*, and take observed values *y_i_*. With *N* hospitals, there are $p_{1},\ldots ,p_{N}$, potentially unique probabilities of treatment. The data can be described by a two-stage sampling model:

(1) $$\begin{array}{c} Y_{i}|p_{i}\overset{indep}{∼}\;\text{Binomial}\;(n_{i},p_{i})\\ p_{i}|F_{p}\overset{iid}{∼}(\cdot |\eta ) \end{array}$$

The hospital-specific parameter *p_i_* comes from a population distribution, *F_p_*, that depends on parameters η. Let *f_p_* be the probability density function (PDF) associated with *F_p_*. We are interested in estimating *f_p_* and its variance $\sigma_{p}^{2}=\int (u-\mu_{p}{)}^{2}f_{p}(u)\text{d}u$, where *μ_p_* = $\int uf_{p}(u)\text{d}u$.

As noted by Paddock and Louis,^19^ there are applications where the observed *N* hospitals constitute the full population of interest. In that case, one is interested in the finite probabilities $p_{1},\ldots ,p_{N}$, with variance $\sigma_{N}^{2}$ = $\frac{1}{N}\sum_{i=1}^{N}(p_{i}-\bar{p}{)}^{2}$ where $\bar{p}=\frac{1}{N}\sum_{i=1}^{N}p_{i}$, and empirical distribution function (EDF), $F_{N}(x)$ = $N^{-1}\sum_{i=1}^{N}I_{\left(p_{i}\leq x\right)}$. If $p_{1},\ldots ,p_{N}$ were observed, one might calculate $\sigma_{N}^{2}$ and *F_N_* as consistent estimators for $\sigma_{p}^{2}$ and *F_p_*. Here, we focus on the former, population-level inference, and address the problem that $p_{1},\ldots ,p_{N}$ are not observed. In accordance with the CRUSADE early follow-up example, our conclusions about variability are meant to apply to hospitals from a broader population, not limited to those participating in the registry.

### 2.1 Raw proportions

As described above, a standard approach to this problem is to obtain sample proportions for each hospital as p^i=yi/ni. The sample variance of p^i is σp^2=1N∑i=1N(p^i-^¯p)2 where ^¯p=1N∑i=1Np^i. The EDF is defined as Fp^(x)=N-1∑i=1NI(p^i≤x), and the distribution of p^1,…,p^N is converted to a smooth density using a kernel density estimate (KDE) with the default bandwidth in the R density() function, denoted fp^.

### 2.2 Gaussian hierarchical model

The generic two-stage model for *Y_i_* is provided in Equation (1). Here, we focus on the Gaussian, logistic model by adding parametric assumptions. The model assumes:

(2) $$\begin{array}{c} Y_{i}|p_{i}\overset{indep}{∼}\;\text{Binomial}(n_{i},p_{i})\\ \text{logit}(p_{i})=\beta_{0}+b_{i}\\ b_{i}|\sigma_{b}^{2}\overset{iid}{∼}\text{Normal}(0,\sigma_{b}^{2}) \end{array}$$

where logit(*x*) = log{x/(1-x)}. A common goal is to estimate $\beta_{0}$ and $\sigma_{b}^{2}$, while acknowledging *b_i_* as a source of variation; *b_i_* is integrated out of the likelihood, and not directly estimated as part of the model fit. When there is interest in estimating or predicting *p_i_* (as a function of *b_i_*), a variety of approaches can be implemented in a second phase of estimation.^20,21^ An empirical Bayes (EB) approach is widely utilized for this purpose.^8,20^ We denote the EB predictions as p^ib. The variance of p^1b … p^Nb,σp^b2 is over-shrunk compared to $\sigma_{p}^{2}$. Although this fact is well known, EB predictions are nearly synonymous with hierarchical models in cardiovascular research. To emphasize the difference between EB prediction and the following approach, σp^b2 is included in our simulation study of provider-level variance.

For the current application, we are not interested in the individual *p_i_*, but in describing their distribution. Given that logit(*p*) is assumed to follow a normal distribution, Normal$\left(\beta_{0},\sigma_{b}^{2}\right)$, we can estimate the parameters of this distribution, plug them in, and parametrically estimate $\sigma_{p}^{2}$ and *f_p_*. Implementation is as follows. Using standard statistical packages, fit the hierarchical model to obtain estimates of $\beta_{0}$ and $\sigma_{b}^{2}$. Substitute estimates for the unknown parameters and generate *q* = logit(*p*) according to the normal distribution, Normal(β^0,σ^b2). This can be done either by simulation or by applying the normal quantile function to a sequence of uniform probabilities between 0 and 1. Then, convert to the probability scale by taking $\text{exp}(q)/\{1+\text{exp}(q)\}=\overset{∼}{p}$. By taking a large number of data points *q*, and therefore $\overset{∼}{p}$, the density of these data will be arbitrarily close to the density of $\overset{∼}{p},f_{\overset{∼}{p}}$, which is regarded as an estimator of *f_p_*. The variance of $\overset{∼}{p},\sigma_{\overset{∼}{p}}^{2}$, is an estimator of $\sigma_{p}^{2}$. Alternative methods could be used to derive $f_{\overset{∼}{p}}$, such as the transformation theorem applied to the function exp(q)/{1+exp(q)}. This would also have to be obtained numerically.

Clearly, $f_{\overset{∼}{p}}$ is derived under the assumption that *q* has a normal distribution. In many cases, this is a reasonable assumption. As with many natural phenomenon, it is plausible that hospitals would have symmetric variation about a common mean on the log-odds scale. The Gaussian hierarchical model (GHM) approach, just described, illustrates the result on a clinically meaningful scale. If normality is not plausible, estimating provider variation may still be insensitive to such mis-specification, as previous authors have observed robust results, even when the random effects distribution is mis-specified.^22^ The robustness of the variance parameter to violations of the Gaussian assumption is explored below.

### 2.3 Semi-parametric Bayesian density estimation

Bayesian methods offer flexibility in the specification of hierarchical models and density estimation. Rather than specifying a parametric prior distribution for cluster-specific probabilities, the Dirichlet process (DP) prior has been widely used for parametric mixture modeling.^11,12,14,15,17,23^ Numerous options and software are reviewed by Jara et al.^23^ The DPs mixture can approximate many smooth distributions by estimating the number of mixture components according to the data. One such model, based on the methods of Escobar and West^14^ and Liu,^17^ is implemented by the DPbetabinom function in R DPpackage.^23^ The function fits a semi-parametric version of the beta-binomial model

$$\begin{array}{c} Y_{i}|p_{i}\overset{indep}{∼}\;\text{Binomial}(n_{i},p_{i})\\ p_{i}|F_{p}∼F_{p}\;j=1\ldots N\\ F_{p}|\alpha ,F_{0}∼\text{DP}(\alpha F_{0})\\ F_{0}=\text{Beta}(a_{1},b_{1})\\ \alpha |a_{0},b_{0}∼\text{Gamma}(a_{0},b_{0}) \end{array}$$

For this study, we assume $F_{0}∼\text{Uniform}(0,1)$, i.e., $a_{1}=1,b_{1}=1$. The baseline distribution, *F*_0_, is conjugate in this model which allows Markov chain Monte Carlo with Gibbs sampling for estimating the posterior density $f_{p|Y}$. The posterior variance is defined as $\sigma_{p|Y}^{2}$ = $\int (u-\mu_{p|Y}{)}^{2}f_{p|Y}(u)\text{d}u$, where $\mu_{p|Y}$ = $\int uf_{p|Y}(u)\text{d}u$. The posterior density $f_{p|Y}$ and $\sigma_{p|Y}^{2}$ are estimators of *f_p_*, and $\sigma_{p}^{2}$, respectively.

In preliminary studies, we evaluated potential values for the hyper-parameters *a*_0_ and *b*_0_ or the prior parameter *α* (removing the hyper-prior). Plausible values for *α* range from 1/logN to N/logN (0.1 to 80 in the simulation settings below).^11^ When *α* is near 0, the model places high probability on *p_i_* being all the same, and the posterior density tends to be highly peaked around finite points. As *α* increases, this leads to a higher probability of more unique values of *p_i_*, and the posterior density tends to be more smooth. Excessively large *α* will treat each provider probability as if they are totally unique and tend toward a fixed effects model. The gamma hyper-prior is employed to increase robustness by allowing *α* to be better adapted to the data. We considered α∈{0.1,1,5,10,20,50} and multiple sets of hyper-parameters for the gamma prior on *α*. None performed better than those of Paddock et al.,^15^ $a_{0}=4$ and $b_{0}=4$ (subsequently denoted DP-1) and $a_{0}=10$ and *b*_0_ = 0.10 (subsequently denoted DP-2). We therefore focus on this specification in the subsequent evaluation. Given a sample of 100 hospitals, 95% of the prior mass on the number of clusters in the DP mixture for these two pairs of hyper-parameters falls on 10 or fewer clusters (DP-1) and between 53 and 83 clusters (DP-2). Results for a fixed *α* = 20 are also displayed as DP-3.

### 2.4 Smoothing by roughening

Rather than specifying a parametric prior distribution for the provider-specific parameters, Laird^24,25^ developed a non-parametric maximum likelihood (NPML) estimator of the prior, *F_p_*. Despite numerous advantages, this approach yields discrete priors with too narrow support and under-dispersion,^13^ motivating the adaptation of a non-parametric EB alternative called “smoothing by roughening” (SBR).^13,26^ The process starts with a smooth prior distribution, that is not necessarily correct, and uses the EM algorithm to update it in the direction of the NPML. Fewer iterations results in a more smooth distribution, while increasing iterations are successively closer to the NPML. To speed up computation, the initial smooth density is discretized at a large number of mass points **a** = $(a_{1},\ldots ,a_{M}{)}^{T}$, with initial probabilities $f_{0}(a)=[f_{0}(a_{1}),\ldots ,f_{0}(a_{M}){]}^{T}$. The distribution of **a** is updated over $\nu =1,\ldots ,\nu_{N}$ steps, as follows:

(3) $$\begin{array}{c} f_{\nu +1}(a_{m})=\frac{1}{N}\sum_{i=1}^{N}f_{\nu }(a_{m}|Y_{i})=\frac{1}{N}\sum_{i=1}^{N}\frac{f_{\nu }(a_{m},Y_{i})}{f_{\nu }(Y_{i})} \end{array}$$

for m=1,…,M. This can be expanded further as

(4) $$\begin{array}{c} f_{\nu +1}(a_{m})=\frac{1}{N}\sum_{i=1}^{N}\frac{f(Y_{i}|a_{m})f_{\nu }(a_{m})}{\sum_{m=1}^{M}f(Y_{i}|a_{m})f_{\nu }(a_{m})} \end{array}$$

where $f(Y_{i}|a_{m})=$ Binomial$\left(n_{i},a_{m}),f_{\nu }(Y_{i})=\sum_{m=1}^{M}f(Y_{i}|a_{m})f_{\nu }(a_{m}\right)$ and $f_{\nu }(a_{m},Y_{i})=f(Y_{i}|a_{m})f_{\nu }(a_{m})$. The initial density *f*_0_ is taken to be Uniform(0, 1). After *ν_N_* iterations, the algorithm stops, and $f_{\nu_{N}}$ is the SBR estimator of *f_p_*. The posterior variance, defined as $\sigma_{\nu_{N}}^{2}$ = $\int (u-\mu_{\nu_{N}}{)}^{2}f_{\nu_{N}}(u)\text{d}u$, where $\mu_{\nu_{N}}$ = $\int uf_{\nu_{N}}(u)\text{d}u$, is the SBR estimator of $\sigma_{p}^{2}$.

When iterated to convergence, the SBR algorithm produces the NPML estimate; however, the goal of SBR is to produce a smooth distribution by the selection of finite *ν_N_*. The appropriate choice of *ν_N_* is unclear, though Shen and Louis^13^ provide some guidance. They suggest that “There is no need to be too strict on the exact value of *ν_N_*, as long as for large N, *ν_N_* is of order log *N*, … For small or moderate samples, we recommend $N/3\leq \nu_{N}\leq 2N$.” In the sample sizes considered below, log *N* would be about 6, whereas N/3 would range from 30 to 150, and 2*N* would range from 200 to 1500. Several values of *ν_N_* are compared in online Supplemental Appendix E. We selected $\nu_{N}=50$ as it yielded superior results in a variety of cases, but no value of *ν_N_* was observed to be universally preferable.

### 2.5 Moment-adjusted imputation

By viewing this as a measurement error problem, additional options become available. Thomas et al.^18^ introduced moment-adjusted imputation (MAI) which replaces mis-measured data, *W*, with estimators that have asymptotically the same distribution as a latent variable of interest, *X*, up to some finite number of moments. They show that MAI is related to other measurement error methods but has superior performance in many settings. MAI is applicable whenever the error in *W* is due to added noise; *W_i_* = *X_i_* + *U_i_*, where *U_i_* is normally distributed random error with mean 0 and variance $\sigma_{\mathrm{ui}}^{2}$. This type of error arises as a result of device error or biological fluctuations but also by random sampling error in parameter estimates. To see that MAI applies to the present application we can view *p* as the unobserved latent variable (*X*) and p^ as a mis-measured version (*W*). p^i can be written as *p_i_* + *U_i_* where *U_i_* is a random error with mean 0 and variance $\sigma_{\mathrm{ui}}^{2}$ = $p_{i}(1-p_{i})/n_{i}$. For hospitals with a reasonably large number of patients, the error in p^i is approximately normal. When the number of patients per site is small, this approximation may not hold. The performance across a range of finite site sizes is evaluated by the simulation given below.

The details of MAI have been described previously, with application to a broad variety of problems.^18^ Here, we review a special case of MAI that is applicable to the current focus on cluster-specific proportions. For the clarity of exposition, we use general MAI notation in this section and refer to *X* and *W*. The objective is to construct-adjusted versions of the *W_i_*, say X^i, where the first *M* sample moments of X^i unbiasedly estimate the corresponding moments of *X_i_*; that is, E(N-1∑i=1NX^ir) = $E(X^{r}),r=1,\ldots ,M$. The distribution of X^i approximates that of *X_i_* up to *M* moments. In particular, the variance of X^i will be unbiased for the variance of *X_i_*. Unbiased estimates, m^r, for the moments, $E(X^{r})$, are derived according to the assumption of normal, additive error, and variance $\sigma_{\mathrm{ui}}^{2}$. The adjusted X^i is obtained by minimizing $\sum_{i=1}^{N}(W_{i}-X_{i}{)}^{2}$ subject to constraints on the moments. Using Lagrange multipliers $(\lambda_{1},\ldots ,,\lambda_{M})=\Lambda$, the objective function is

QM(X1,...,Xn,Λ)=N−1∑i=1N12(Wi−Xi)2+∑r=1Mλrr(N−1∑i=1NXir−m^r)

The adjusted data are obtained by taking the derivative of *Q_M_* with respect to $\left(X_{1},\ldots ,X_{N},\Lambda \right)$, equating this to 0, and solving for (X^1,…,X^N,Λ^) by the Newton–Raphson method. This process has been integrated into an R function (online Supplemental Appendix A). Once the adjusted data X^i are obtained, they can be “imputed” in place of *X_i_*. In particular, the data points X^i can be displayed in a histogram or kernel density plot and will resemble the distribution of *X_i_* up to *M* moments. At a minimum, the mean and variance of X^i will be unbiased for *X_i_* when M≥2.

For the current application, MAI can be applied directly to the “mis-measured” p^i, which takes the place of *W_i_*, and the measurement error variance estimated by σ^ui2 = p^i(1-p^i)/ni. Adjusted values, p^MAI, will have *M* moments that are consistent for the corresponding moments of *F_p_*, as both *n_i_* and *N* get large. The sample variance, σp^MAI2, EDF, Fp^MAI, and density fp^MAI are calculated as for p^, but substituting adjusted values p^MAI.

Thomas et al.^18^ provide an extensive simulation study of MAI, with application to kernel density estimation. They recommend matching an even number of moments (two or four). When *p* has a normal distribution, it is adequate to match two moments, when *p* has a Chi-square or bi-modal distribution, it is necessary to match four moments in order to capture unique features. The tradeoff between matching two versus four moments is a bias-variance tradeoff. Higher order moments are estimated with less precision, and MAI uses estimated moments. Matching higher moments reduces bias but adds variation. Thomas et al.^18^ show that matching four moments strikes a good balance in many reasonable scenarios. As the advantage of MAI is the flexible handling of non-normal distributions, a general recommendation is to match four moments.

### 2.6 Small sites

From a subject matter standpoint, excluding hospitals with very few eligible patients is clinically reasonable; researchers do not expect to characterize a hospital based on a handful of patients. The ad hoc convention is to exclude *n_i_* < 25. Although this exclusion is insufficient, it can be relaxed when the methods considered here are employed. However, there is a limit to the feasibility of recovering *f_p_* when hospital sizes become small. Effectively, the ratio of noise to signal becomes very large; a scenario in which *f_p_* cannot be identified without strong parametric assumptions.^27^ Thus, we do not expect good performance of non-parametric or semi-parametric methods for the estimation of *f_p_* with extremely small hospital sizes. Preliminary simulations indicate that a minimum $n_{i}>10$ and median *n_i_* > 20 is sufficient to avoid numerical problems and poor performance. We focus our attention on applications such as CRUSADE where it is reasonable to require *n_i_* > 10, and the typical cluster size is at least 20. Even so, we consider cluster sizes that are smaller than have been previously evaluated (to the best of our knowledge). A recent tutorial on Bayesian methods for a similar application (hospital comparisons) excluded sites with less than 20 patients (median *n_i_* = 295).^12^ Another example focused on even larger clusters (cities).^13^

## 3 Simulation studies

In this section, we compare the preceding methods by simulation across a range of conditions, including (1) number of providers *N*: 100, 300, and 500; (2) number of patients per provider *n_i_*: 20, median ≈25, 30, median ≈65; (3) distribution of logit $\left(p_{i}\right)$ (i.e., *b_i_* in Equation (2)): Normal$\left(0,\sigma_{b}^{2}\right)$; $\chi_{\mathrm{df}=1}^{2}$, standardized to have mean 0 and variance $\sigma_{b}^{2}$; and a bi-modal mixture of normals; and (4) variation across providers $\sigma_{b}^{2}$: 0.05 (small) and 0.50 (large). Two settings of *n_i_* involve heterogeneous patients per site, taking values similar to those observed in two databases. Histograms of *n_i_* are available in online Supplemental Appendix B. The first represents a distribution of *n_i_* from the CRUSADE early follow-up study which, as a large registry, tends to have larger numbers of patients per site (median ≈ 65, IQR: 38–126). The second replicates the distribution of site sizes from a recent clinical trial, more heavily weighted toward a small number of patients (median ≈ 25, IQR: 17–43). In both, we have excluded sites with $n_{i}<10$ (less than 15% of hospitals and less than 1% of patients). In each simulated data set, a sample of *N* provider probabilities, $p_{1},\ldots ,p_{N}$, is generated according to the distributions described above. At each hospital, *n_i_* patient responses are generated according to a Bernoulli distribution with probability *p_i_*.

### 3.1 Illustration

**Figure 2.**
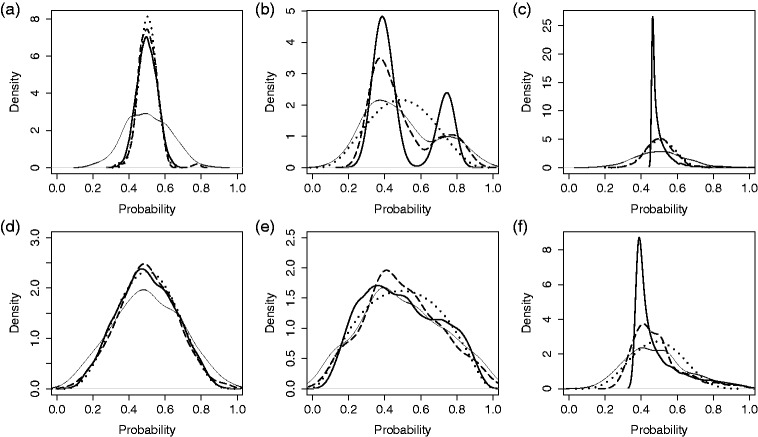
Comparison of methods for estimating the distribution of probabilities across providers. Results from a single, simulated data set for three provider probability distributions and two magnitudes of provider variation: (a-c) small underlying variation, $\sigma_{b}^{2}=0.05$; (d-f) large underlying variation, $\sigma_{b}^{2}=0.5$. Methods: Reference, underlying density of provider probabilities *f_p_* (thick solid line); KDE of raw portions (thin solid line); estimated density from the Gaussian hierarchical model (dotted line); KDE of MAI proportions (dashed line). (a) Normal distribution. (b) Bi-modal distribution. (c) Chi-square distribution. (d) Normal distribution. (e) Bi-modal distribution. (f) Chi-square distribution.

**Figure 3.**
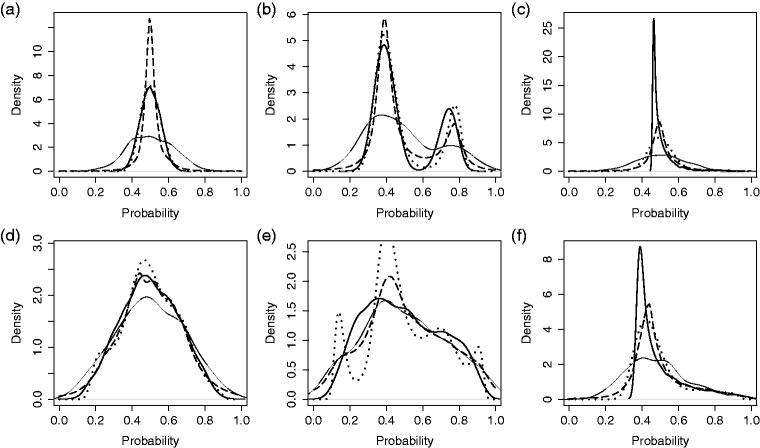
Comparison of methods for estimating the distribution of probabilities across providers. Results from single, simulated data sets with three provider probability distributions and two magnitudes of provider variation: (a-c) small underlying variation, $\sigma_{b}^{2}=0.05$; (d-f) large underlying variation, $\sigma_{b}^{2}=0.5$. Methods: Reference, underlying density of provider-probabilities *f_p_* (thick solid line); KDE of raw portions (thin solid line); smoothing by roughening $f_{\nu_{N}}$ with $\nu_{N}=50$ (dotted line); Bayesian density estimation DP-2 with hyper-prior parameters *a*_0_ = 10 and *b*_0_ = 0.1 (dashed line). (a) Normal distribution. (b) Bi-modal distribution. (c) Chi-square distribution. (d) Normal distribution. (e) Bi-modal distribution. (f) Chi-square distribution.

In Figures 2 and 3, the simulation settings are demonstrated along with select results for single simulated data sets. For this illustrative example, we fix the number of providers at 300 and the number of patients per provider at 20. Densities $f_{\overset{∼}{p}}$ and fp^MAI corresponding to the GHM and MAI are shown in Figure 2, with comparison to *f_p_* and fp^. The densities based on SBR and DP-2 are displayed separately, in Figure 3, although applied to the same data set and compared to the same *f_p_* and fp^. Each density is displayed for both small and large variation across providers. These figures emphasize four things: (1) that our simulation conditions cover a wide range of provider distributions; (2) variation across providers is severely over-estimated by raw proportions when true variation is small, but not when the true variation is large; (3) the adjustment methods can achieve clinically meaningful improvement; and (4) SBR and DP-2 appear to create false modes in some cases. These examples orient us to the simulation settings and illustrate the potential advantages and disadvantages of alternative approaches. A full Monte Carlo (MC) simulation is conducted in order to establish performance over repeated samples and different conditions.

### 3.2 Monte Carlo simulation

**Figure 4.**
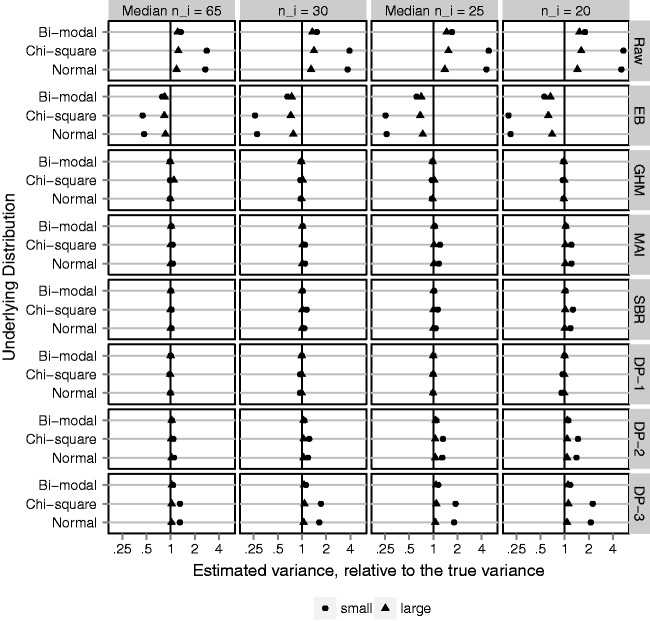
Cluster-level variance estimate; averaged over 1000 simulated data sets and plotted on the log scale, as a ratio, relative to the true variance, $\sigma_{p}^{2}$. Black vertical reference line at 1, indicating no bias in the estimate. Fixing *N* = 300 clusters and varying (1) four cluster sizes, *n_i_*: 20, mixture with median 25, 30, and mixture with median 65; (2) underlying variation: small and large with $\sigma_{b}^{2}=0.05\;\text{ and }\;0.50$, respectively; (3) underlying distribution of cluster-level probabilities: Normal, Chi-square, Bi-modal. Methods: Raw, raw proportions; GHM, Gaussian hierarchical model; EB, empirical Bayes from GHM; MAI, moment-adjusted imputation; SBR, smoothing by roughening; DP-1, Beta-binomial hierarchical model with Dirichlet prior with hyper-prior parameters favoring roughness; and DP-2, same with hyper-prior parameters favoring smoothness; and DP-3, same with fixed prior parameter favoring smoothness.

**Figure 5.**
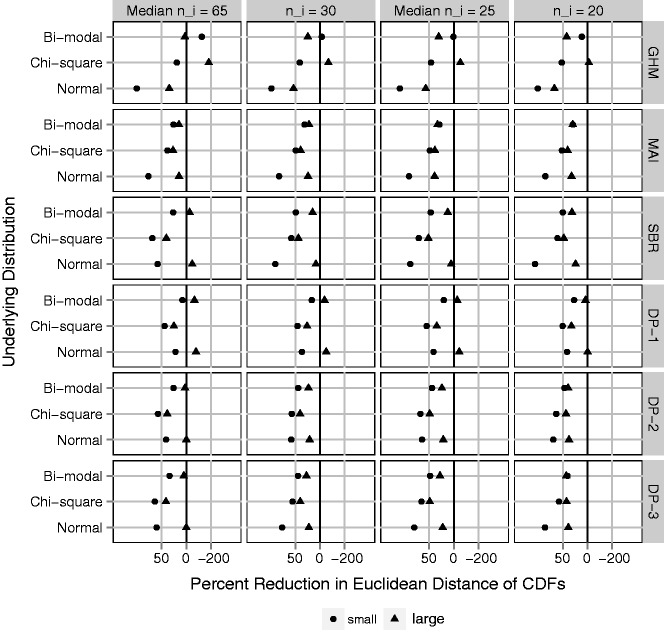
Euclidean distance between estimated distribution functions and the underlying distribution of cluster-level probabilities, *F_p_* (ED-CDF); average over 1000 simulated data sets and plotted on the log scale as a percent reduction relative to the ED-CDF for raw proportions (naive method). Black vertical line represents no difference. Gray vertical lines represent 50% improvement (left) and 200% worse (right). Fixing *N* = 300 clusters and varying (1) four cluster sizes, *n_i_*: 20, mixture with median 25, 30, and mixture with median 65; (2) underlying variation: small and large with $\sigma_{b}^{2}=0.05\;\text{ and }\;0.50$, respectively; (3) underlying distribution of cluster-level probabilities: Normal, Chi-square, Bi-modal. Methods: GHM, Gaussian hierarchical model; MAI, moment-adjusted imputation; SBR, smoothing by roughening; DP-1, Beta-binomial hierarchical model with Dirichlet prior with hyper-prior parameters favoring roughness; and DP-2, same with hyper-prior parameters favoring smoothness; and DP-3, same with fixed prior parameter favoring smoothness.

**Figure 6.**
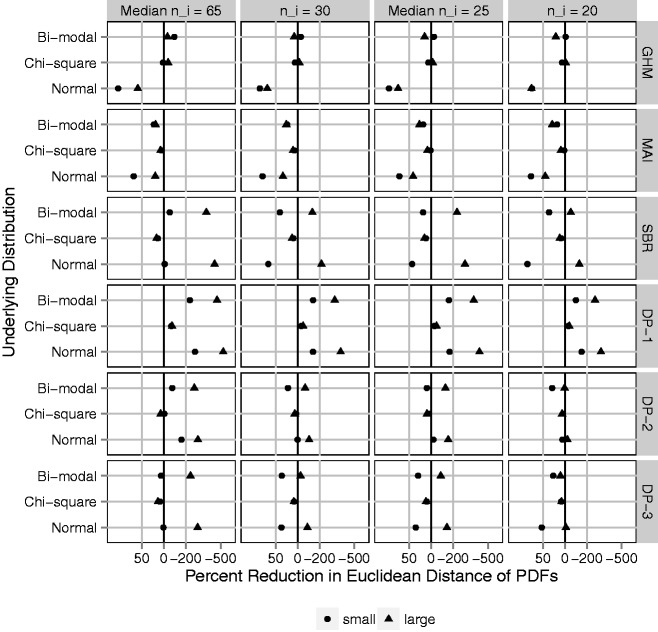
Euclidean distance between estimated density function and the underlying distribution of cluster-level probabilities, *f_p_* (ED-PDF); average over 1000 simulated data sets and plotted on the log scale as a percent reduction relative to the ED-PDF for raw proportions (naive method). Black vertical line represents no difference. Gray vertical lines represent 50% improvement (left) and 200% worse (right). Fixing *N* = 300 clusters and varying (1) four cluster sizes, *n_i_*: 20, mixture with median 25, 30, and mixture with median 65; (2) underlying variation: small and large with $\sigma_{b}^{2}=0.05\;\text{ and }\;0.50$, respectively; (3) underlying distribution of cluster-level probabilities: Normal, Chi-square, Bi-modal. Methods: GHM, Gaussian hierarchical model; MAI, moment-adjusted imputation; SBR, smoothing by roughening; DP-1, Beta-binomial hierarchical model with Dirichlet prior with hyper-prior parameters favoring roughness; and DP-2, same with hyper-prior parameters favoring smoothness; and DP-3, same with fixed prior parameter favoring smoothness.

In *B* = 1000 simulated data sets, a sample of *N* providers is drawn, with probabilities of outcome generated according to the distributions for logit(*p*) described above. For each data set and method, we obtain estimates of $\sigma_{p}^{2}$, *F_p_*, and *f_p_*. Success in estimating the provider-level variance is measured by bias and mean squared error (MSE). Bias is quantified by comparing the MC average of provider-level variance estimates, 1B∑bB{σ^p,b2}, to the true $\sigma_{p}^{2}$. The MSE is given by 1B∑bB{σ^p,b2-σp2}2. The Euclidean distance (ED) is calculated between cumulative distribution function estimates and *F_p_* (ED-CDF), as well as between PDF estimates and *f_p_* (ED-PDF). In a single data set ED-CDF = ∫{F^(t)-Fp(t)}2dt and ED-PDF = ∫{f^(t)-fp(t)}2dt, where F ^ and f ^ are replaced by the estimate for each method. Results are shown in Figures 4 to 6 for *N* = 300 providers. Relative performance did not vary substantially based on *N*. Full results are in online Supplemental Appendix C.

Figure 4 shows the MC average provider-level variance estimates plotted as a ratio, relative to the true variance, on the log scale. The ratio is taken so that different underlying distributions and magnitudes of variance can be plotted on a common scale. Ratios less than 1 reflect estimators that under-estimate the variance, whereas ratios greater than 1 reflect estimators that over-estimate the variance. The log scale is used so that a 50% under-estimate is the same distance from 1 as a 200% over-estimate. Figure 4 shows that the naive method, based on raw proportions, substantially over-estimates the variance. As expected, the Gaussian EB predictions are just as bad, in the opposite direction. The candidate adjustment methods, GHM, MAI, SBR, and DP-1, are almost perfectly unbiased. Of note, the GHM variance estimator is unbiased even when the underlying distribution is chi-square or bi-modal and the Gaussian assumption is incorrect. However, the Bayesian semi-parametric method is sensitive to the specification of prior parameters; priors DP-2 and DP-3 slightly over-estimated the cluster-specific variance, particularly when the underlying variation is small. Results for the MSE track closely with bias and are reported in online Supplemental Appendix C. Adjustment methods reduced the MSE, relative to raw proportions, by 60% to 99%.

The ED provides a global measure of closeness between the estimated distributions and their target. Figure 5 shows the average over simulated data sets of ED-CDF for each adjustment method, plotted as a percent reduction relative to the average ED-CDF for raw proportions. The goal is to quantify improvement in estimating *F_p_* compared to taking a naive approach. MAI is the only adjustment method that reduces the ED-CDF in all scenarios. As expected, GHM does best when the underlying distribution function is Gaussian, but poorly otherwise. SBR performs similar to MAI, but in a few cases increases the ED-CDF. Investigation of individual data sets (online Supplemental Appendix D) reveals that this can be attributed to SBR identifying too many peaks or multiple modes when the target distribution is smooth. Among the DP methods, DP-2 and DP-3 perform better than DP-1. This makes sense considering that DP-2 and DP-3 favor a smooth distribution, whereas DP-1 identifies too many peaks and modes rather than a smooth distribution (online Supplemental Appendix D). The many peaks have minimal impact on the variance estimate but matter with respect to estimating *F_p_*.

The ED-CDF metric does not tell the full story. Provider variation is frequently illustrated by plotting a density estimate, which may also be of interest. Even if the primary goal focuses on variation, a density estimate with multiple modes or unique skew is likely to attract attention. Therefore, accuracy for density estimation is important. Figure 6 shows the average over simulated data sets of ED-PDF, plotted as a percent reduction relative to the average ED-PDF for raw proportions. On this metric, improving density estimation relative to raw proportions is not guaranteed by any adjustment method. As expected, the GHM provides large improvements only if the underlying distribution is Gaussian. Otherwise it is generally worse. MAI improves the ED-PDF as much as 25% to 75% when the underlying distribution is normal or bi-modal, though very little when the underlying distribution is Chi-square. In online Supplemental Appendix D, MAI produces a rare outlier in the case of small *n_i_* and small, chi-square provider variation. The outlier was easily recognizable for having mass on a few discrete points and may have skewed the average performance in this scenario. This never occurred with larger *n_i_* (≥30), regardless of the underlying distribution. SBR and DP-1 increased the ED-PDF by as much as 500% in many cases. The problem was most severe in large variation scenarios, worsened with increasing cluster size *n_i_*, and did not improve with increasing *N*. Investigation of individual data sets (online Supplemental Appendix D) reveals density estimates with 10 to 20 modes, when the underlying distribution had only one or two. DP-2 and DP-3 favor a more smooth distribution and yet the same problem was apparent.

## 4 Analysis of early physician follow-up

**Figure 7.**
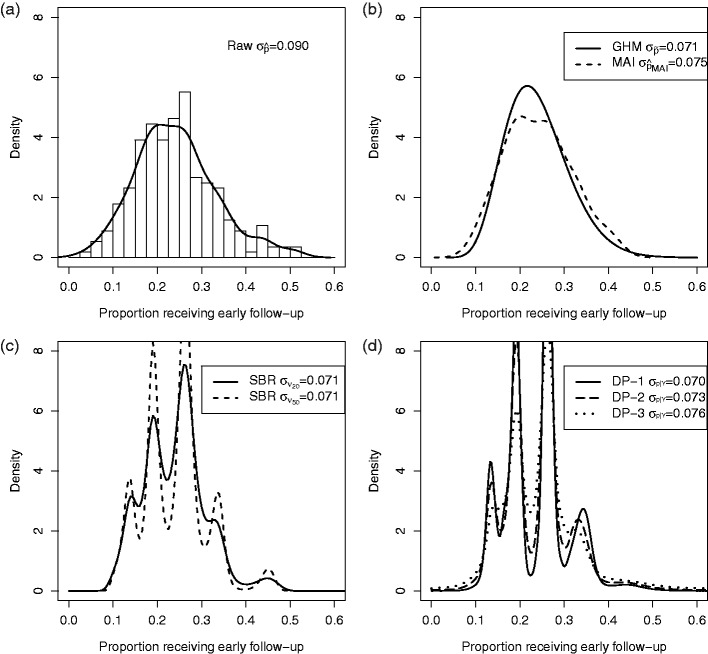
Variation in early follow-up across providers. (a) Density of raw proportions. (b) Estimated densities by GHM and MAI. (c) Estimated densities by SBR. (d) Estimated densities by DP priors. DP: Dirichlet process; GHM: Gaussian hierarchical model; MAI: moment-adjusted imputation; SBR: smoothing by roughening.

To demonstrate these methods, we revisit the example of early physician follow-up described in Section 1. Using the raw proportions, calculated within each provider, Hess et al.^5^ saw wide variation in early follow-up with a physician after MI (Figure 7(a)). In Figure 1, we re-analyze the CRUSADE data and directly estimate the density of the provider-specific proportion of patients receiving early follow-up using a GHM, MAI, SBR, and DP methods. In Figure 7(c), SBR is displayed for $\nu_{N}=20$ and 50, in order to visualize the sensitivity to the choice of iterations. Each of the DP priors is included in Figure 7(d).

Comparing Figure 7(a) with 7(b) to (d), the standard deviation is smaller when chance variations are removed. The adjusted distributions in Figure 7(b) to (d), have nearly identical spread. Compared to GHM, MAI preserves some unique features of the raw distribution, such as slight bi-modality near the peak, whereas SBR and DP methods create clusters of provider probabilities. Our simulation studies suggest that those clusters may be false and occur even if the underlying distribution of provider probabilities is smooth. Figure 7(b) to (d) conveys the same essential information that the wide variation in the use of early follow-up observed by Hess et al.^5^ was largely reflective of real variation.

## 5 Discussion

When the goal of an analysis is to estimate the magnitude of systematic differences among providers, the random variation associated with observed rates muddies the picture. We illustrate the use of existing methods for this purpose and assess their merits under settings that are representative of hospital variation studies, including smaller cluster sizes than have previously been evaluated. Further, we demonstrate a novel application of MAI. Our results emphasize that each has limitations. The choice of methods should be guided by the priorities of a given study and the available data.

When the goal is to quantify cluster-level variation and flexible density estimation is not a priority, the GHM performs well; its variance is unbiased regardless of whether the underlying distribution is Gaussian. This result does not depend on the number of hospitals, *N*, or hospital sizes, *n_i_*. The GHM is particularly useful if the number of providers is small (N≤100) and/or the number of patients per provider is small (median $n_{i}\leq 25$), but the parametric assumption imposed by the model must be acknowledged. If flexible density estimation is desired, larger site sizes are needed. Otherwise all of the methods considered here have potential problems. With median $n_{i}\leq 25$, MAI may produce densities with mass on a few discrete points; while exceedingly rare, this cannot be ruled out without the knowledge of the underlying density. With hospital sizes larger than 30, MAI performs well, whereas SBR and DP methods estimate incorrect multi-modal densities. The gains in density estimation from MAI are modest, with a reduction in ED-PDF of 0 to 75%. However, this compliments a large improvement in variance estimation (60%–95% reduction in MSE of the variance).

Although DP-1, DP-2, and DP-3 reduce bias in the estimation of provider-level variability, the resulting densities are not accurate. Given that our motivating applications seek to illustrate variability via a density estimate, this method could be misleading. The current analysis is based on a mixture of DPs that has been proposed for the purpose of flexible density estimation and incorporated into software that is apparently straightforward to use.^14,17,23^ Our specification of hyper-prior parameters is consistent with the previous literature,^15^ and DP-1 is similar to fixing *α* = 1, which is default in the documentation of DPbetabinom(). However, the problems we observe are not easily resolved by modifying the hyper-prior parameters. We explored a large number of hyper-prior parameters prior to focusing on DP-1, DP-2, and DP-3. Alternatives were not superior. Therefore, our results are likely representative of a typical application of this software. This does not implicate all Bayesian methods; a Bayesian analysis with Gaussian prior would be nearly identical to the GHM. However, the Bayesian semi-parametric model based on a DP prior was frequently worse than naive methods at estimating the provider-level density. This method does not appear suitable to hospital variation studies that are similar to the settings we evaluate.

SBR exhibits some of the same problems as DP methods. Even with a large number of hospitals and many patients per hospital, the SBR density estimate is often worse than the naive method, with false multi-modality. Shen and Louis^13^ noted that the SBR estimate of *F_p_* behaves similarly to the one based on the DP hyper-prior, depending on the number of iterations *ν_N_*. SBR converges to the nonparametric maximum likelihood estimator (NPML) as the number of iterations increases. The NMPL has been criticized for being discrete, and frequently under-dispersed. Therefore it is not surprising that SBR would exhibit similar features at some number of iterations. Using fewer iterations of SBR is an option. Smaller *ν_N_* will create more smoothing and avoid multi-modality. However, the tradeoff, at least with an uninformative prior, is that the results remain over-dispersed. This phenomenon can be seen in Supplemental Appendix E.

These problems observed with DP and SBR methods were not observed in previous studies that focused on continuous outcomes, larger cluster sizes and estimation of CDFs. In these cases, where there is more information in the data, results would not be as sensitive to choice of prior parameters or iterations. In addition, focusing on the CDF (Figure 5) does not emphasize a problem because the ED-CDF (Figure 5) is insensitive to false modes, since it is primarily driven by differences in center and spread. One advantage to SBR is that it is easy to investigate the sensitivity of results to the choice of *ν_N_*. By saving the output at each step, one can plot the results across a range of *ν_N_*. If the results were insensitive that might provide an additional level of confidence. In our settings, the results were very sensitive. Therefore, there is a high risk of inaccurate results or the potential to pick and chose among them.

Compared to the DP and SBR methods, MAI does not involve decisions regarding hyper-prior or tuning parameters. MAI does not directly target density estimation but “adjusts” the observed data to have unbiased moments up to a fixed number. However, the choice of how many moments to match is analogous to a tuning parameter. We implemented MAI, as proposed by Thomas et al.,^18^ by fixing the number of unbiased moments at four (mean, variance, skewness, and kurtosis). If the number of moments matched were increased, the results would vary. For our purpose, MAI may be preferable because the decision regarding moments is easily interpreted and related to the goals. In order to visualize variability across providers, without any strong parametric assumptions, it may be adequate to have unbiased mean, variance, skewness, and kurtosis. Indeed, this approach performed well across a range of scenarios.

There are a variety of opportunities for extensions and future research. For clarity, we have focused on the example of variation in early follow-up across hospitals, but the same issues and methods apply to any binary outcome and across different types of provider-based clusters (site, region, and physician). Occasionally, the goal is to describe variation across providers, after adjusting for patient characteristics. It is relatively simple to adapt MAI to allow for case-mix adjusted rates, as MAI starts with a set of noisy estimates (potentially adjusted) and removes the noise. However, there are a lot of ways in which adjustment can be defined in terms of the target of inference. This is more complicated because the variation in proportions depends on the mean; different methods of adjustment that alter the center of the distribution may target different variance parameters. In addition, one might consider whether fixed provider-level information could be used to augment the adjustment process. Provider volume, for example, is often of interest. MAI was developed to target the joint distribution of multiple variables including mis-measured and error-free variables. Moments and cross-products can be targeted to achieve this end. MAI could be adapted to remove measurement error in a volume-specific way; preserving the joint distribution between site-specific performance and volume. These extensions are an important avenue for future research.

Quality improvement studies involve many objectives that are not considered here and excellent resources are available.^6–8^ Different objectives within the same study may require different methods. Here, we provide methods that may complement other analyses. Whenever the magnitude of variability is a key finding, it should be estimated and illustrated unbiasedly.
